# Status and related factors of postoperative recurrence of ovarian endometriosis: a cross-sectional study of 874 cases

**DOI:** 10.1007/s00404-023-06932-x

**Published:** 2023-01-28

**Authors:** Xinchun Yang, Meiru Bao, Tian Hang, Weiwei Sun, Yong Liu, Yanhuan Yang, Yiwei Yu, Tingyu Zhao, Ran Xu, Ruijie Hou, Ruihua Zhao

**Affiliations:** Department of Gynecology, Guang’anmen Hospital, China Academy of Chinese Medical Sciences, Beijing, 100053 China

**Keywords:** Ovarian endometriomas, Recurrence, Risk factors

## Abstract

**Purpose:**

Exploring the status and related factors of postoperative recurrence of ovarian endometriosis.

**Methods:**

This study analyzed the results of questionnaires conducted in 27 hospitals across the country from January 2019 to November 2021. All women were divided into recurrence group and non-recurrence group to analyze the recurrence rate and related factors after ovarian endometriosis surgery.

**Results:**

The recurrence rates of ovarian endometriosis within 1 year, 1–2 years, 2–3 years, 3–4 years, 4–5 years and more than 5 years were 6.27%, 35.85%, 55.38%, 65.00% and 56.82%, respectively. Significant differences were found between two groups in terms of age at surgery (OR: 0.342, 95%CI: 0.244–0.481, *P* < 0.001), presence of dysmenorrhea (OR: 1.758, 95%CI: 1.337–2.312, *P* < 0.001), presence of adenomyosis (OR: 1.948, 95%CI: 1.417–2.678, *P* < 0.001) and family history of endometriosis or adenomyosis (OR: 1.678, 95%CI: 1.035–2.721, *P* = 0.021). The age at surgery (OR: 0.358, 95%CI: 0.253–0.506, *P* < 0.001), presence of dysmenorrhea (OR: 1.379, 95%CI: 1.026–1.853, *P* = 0.033) and presence of adenomyosis (OR: 1.799, 95%CI: 1.275–2.537, *P* = 0.001) were significantly associated with endometrioma recurrence in multivariate analysis. No significant associations were found between the recurrence rate and body mass index (BMI), educational background, age of menarche, gravida, parity, uterine leiomyoma, endometrial polyps or postoperative use of gonadotropin-releasing hormone agonist (GnRH-a).

**Conclusions:**

Dysmenorrhea and presence of adenomyosis are independent risk factors for postoperative recurrence of ovarian endometriosis, and older age is an independent protective factor for postoperative recurrence.

## What does this study add to the clinical work

**Table:** 

Dysmenorrhea and presence of adenomyosis are independent risk factors while older age is an independent protective factor for postoperative recurrence of ovarian endometriosis. These findings are instrumental in Long-term management of endometriosis.	

## Introduction

Endometriosis (EM) is a chronic estrogen-dependent disease with endometrioid tissue outside the uterus and affects 5–10% of women of reproductive age [1]. An estimated 176 million women worldwide are affected [2]. The clinical presentations of EM are diverse, including chronic pelvic pain, dysmenorrhea, infertility, fatigue, etc. [3]. The socioeconomic impact of EM is more than 80 billion USD per year, which is similar to diabetes [2]. Long-term pain, infertility, fear of postoperative recurrence and huge economic burden lead to depression and anxiety symptoms of patients, which seriously affect patients' quality of life. Research has found that health-related quality of life was related to mental health status by implementation of EM health profile questionnaire [4, 5].

According to World Endometriosis Society consensus on the classification of EM, three subtypes are described: superficial peritoneal, ovarian, and deep [6]. Ovarian endometriosis is the most common type, which is seen in 17–44% of EM patients [7]. Surgery and medical treatment such as Gonadotropin-releasing hormone agonist (GnRH-a) are important method for the treatment of ovarian endometriosis cysts. In contrast, surgery can not only diagnose endometriosis, but also quickly remove the lesion and relieve symptoms. However, postoperative recurrence rate is high. Among women who have undergone surgery, more than half of them will be re-operated within 5 years [8]. Operation on ovarian endometriomas can result in reduced ovarian reserve, manifested by a decrease in antral follicle count and inhibin B [9, 10]. In addition, surgery may lead to postoperative peritoneal adhesion formation [11]. Therefore, repeat surgery is not recommended. How to inhibit postoperative recurrence so as to avoid repeat surgeries is an important problem to be solved.

At present, there are few large-sample multicenter studies on the recurrence rate and related factors of ovarian endometriosis in China. Therefore, the purpose of this study is to investigate the status and related factors of ovarian endometriosis through cross-sectional investigation.

## Methods

### Participants

We conducted a cross-sectional survey of endometriosis patients in 27 hospitals nationwide from January 2019 to November 2021, and analyzed 874 patients undergoing surgery for ovarian endometriosis diagnosed by pathology.

### Materials

The questionnaire was completed by strictly trained gynecological professionals, including age, height, weight, education level, age of menarche, gravida, parity, presence of uterine leiomyoma, presence of adenomyosis, presence of endometrial polyps, family history of EM/ adenomyosis (AM), postoperative GnRH-a use, presence of dysmenorrhea, time of ovarian endometriosis operation and postoperative recurrence or not and so on. The criteria for postoperative recurrence of ovarian endometriosis was as follows: after the symptoms of endometriosis are relieved by surgical treatment, clinical symptoms reappear, and return to the level before treatment or worsen or endometriotic cyst reappears.

### Statistical analysis

Statistical analysis was performed using the Statistics Package for Social Sciences Version 26.0 (SPSS Inc., Chicago, IL, USA). The counting data were expressed as percentage (%) and χ^2^ test was used. The measurement data were tested for normality, and the data conforming to normal distribution were presented as mean ± standard deviation and *t* test was used. M (QL, QU) was used to represent the non-normal distribution data and nonparametric test was used. Univariate and multivariate logistic regression were used to determine potential risk factors. Odds ratio (OR) and 95% confidence interval (CI) were calculated as measures of recurrence risk. *P* < 0.05 was considered as statistically significant.

## Results

**Fig. 1:**
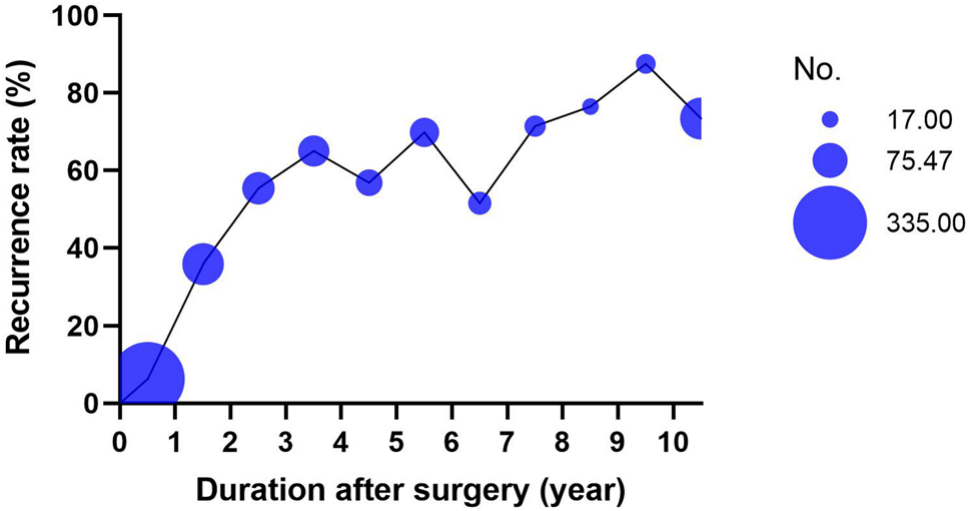
The distribution of the number of people investigated at different time after surgery and the recurrence of ovarian endometriosis

The mean age of 874 women was 35.18 ± 6.187 years, the median postoperative time was 24.00 (6,71) months, 49.77% (435/874) of the patients complicated with dysmenorrhea, 16.70% (146/874) of the patients complicated with uterine leiomyoma, 23.00% (201/874) of the patients complicated with adenomyosis and 499 patients (57.09%) received GnRH-a treatment for 3–6 months. There were 347 women in the recurrent group and 527 women in the non-recurrent group. The recurrence rates of ovarian endometriosis within 1 year, 1–2 years, 2–3 years, 3–4 years, 4–5 years, and more than 5 years were 6.27% 35.85% 55.38% 65.00% 56.82%, respectively (see Table 1 and Fig. 1).

**Table 1:** The distribution of the number of people investigated at different time after surgery and the recurrence of ovarian endometriosis

Time after surgery (year)	Number	Recurrent group (*n* = 347)	Non-recurrent group (*n* = 527)	Recurrence rate %
≤ 1	335	21	314	6.27
1–2	106	38	68	35.85
2–3	65	36	29	55.38
3–4	60	39	21	65.00
4–5	44	25	19	56.82
5–6	53	37	16	69.81
6–7	33	17	16	51.52
7–8	28	20	8	71.43
8–9	17	13	4	76.47
9–10	24	21	3	87.50
> 10	109	80	29	73.39

**Fig. 2:**
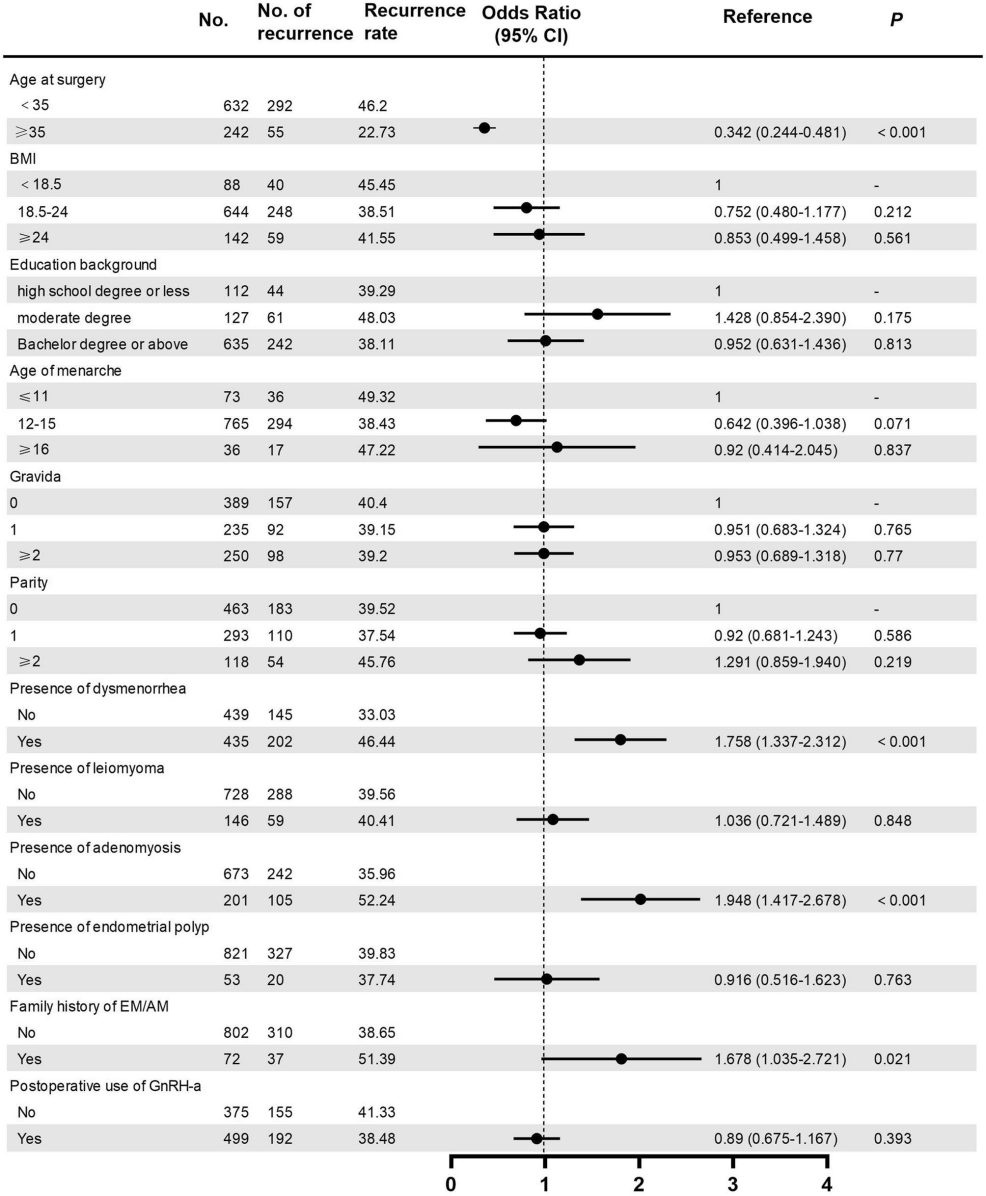
Univariate analysis of risk factors in the endometrioma recurrence and non-recurrence groups. Abbreviations: *BMI* Body mass index, *GnRH-a* Gonadotropin-releasing hormone agonist

**Fig. 3:**
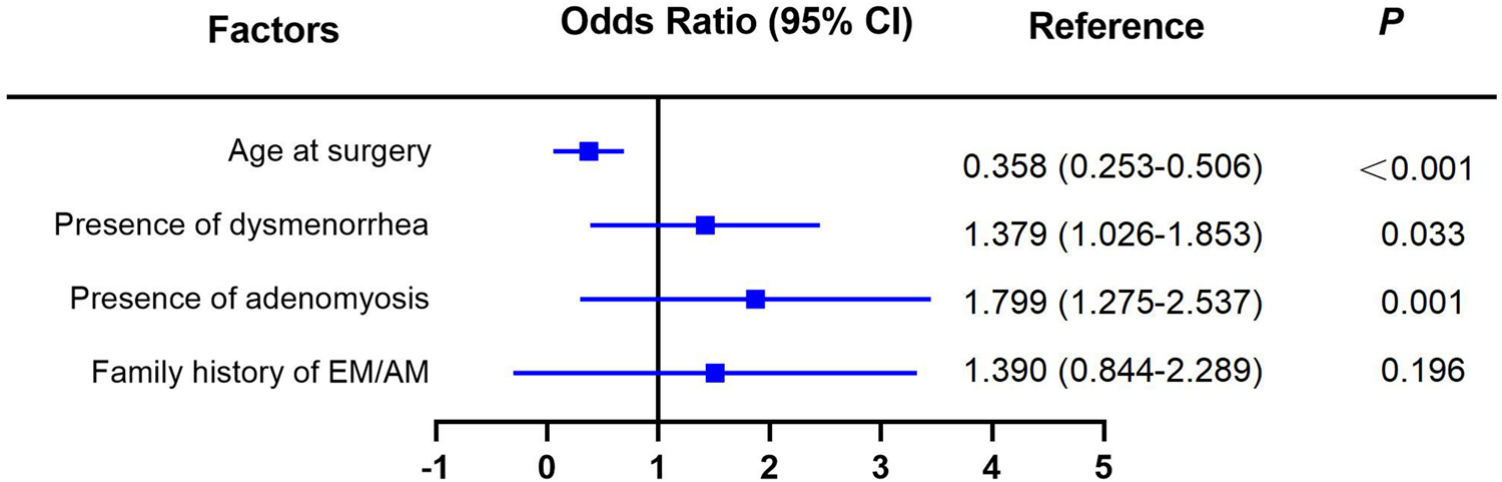
Multivariate analysis of risk factors in the endometrioma recurrence and non-recurrence groups

Univariate and multivariate logistic regression analyses were performed. In univariate analysis, significant differences were found between two groups in terms of age at surgery (OR: 0.342, 95%CI: 0.244–0.481, *P* < 0.001), presence of dysmenorrhea (OR: 1.758, 95%CI: 1.337–2.312, *P* < 0.001), presence of adenomyosis (OR: 1.948, 95%CI: 1.417–2.678, *P* < 0.001) and family history of endometriosis or adenomyosis (OR: 1.678, 95%CI: 1.035–2.721, *P* = 0.021) (see Fig. 2). No significant associations were found between the recurrence rate and BMI, educational background, age of menarche, gravida, parity, presence of uterine leiomyoma, presence of endometrial polyps or postoperative use of GnRH-a. The age at surgery (OR: 0.358, 95%CI: 0.253–0.506, *P* < 0.001), presence of dysmenorrhea (OR: 1.379, 95%CI: 1.026–1.853, *P* = 0.033) and presence of adenomyosis (OR: 1.799, 95%CI: 1.275–2.537, *P* = 0.001) were significantly associated with endometrioma recurrence in multivariate analysis (see Fig. 3).

## Discussion

The aim of this study was to explore the status and related factors of postoperative recurrence of ovarian endometriosis. The results showed that the recurrence rates of ovarian endometriosis within 1 year, 1–2 years, 2–3 years, 3–4 years, 4–5 years, and more than 5 years were 6.27%, 35.85%, 55.38%, 65.00%, 56.82% respectively. We found that dysmenorrhea, presence of adenomyosis and age at surgery were associated with postoperative recurrence of ovarian endometriosis.

As a cross-sectional survey, our study only counted the recurrence status of ovarian endometriosis after surgery. This is different from follow-up studies. It has been reported that the average recurrence rate of EM in 2 years is about 20% ( 0–89.6%), and the recurrence rate in 5 years is as high as 50% (15.1–56%) [12]. Lee et al. [13] reported that the cumulative recurrence rates of 1 year, 2 years, 2.5 years and 5 years after ovarian endometrioma surgery are 3.7%, 6.7%, 11.1%, 16.7%, respectively. In the follow-up of patients with ovarian endometriotic cyst after surgery, Xiao-Yan Li et al. [14] found that the cumulative incidence of recurrence in 5–10 years after surgery was 15.4%, 16.8%, 19.3%, 22.5%, 22.5%, 22.5%, respectively. This may be related to the different definitions of recurrence in the literature and the postoperative treatment of patients.

According to multivariate analysis of our study, the presence of dysmenorrhea is an independent risk factor for postoperative recurrence of ovarian endometriosis. This is consistent with those of several studies [15–17]. In addition, not only the presence but also the severity of dysmenorrhea was an independent risk factor for postoperative recurrence of endometrioma [14]. The mechanism of dysmenorrhea may be related to abnormal innervation and inflammation interaction [18, 19]. Study has found that bradykinin (BK) is involved in the occurrence of EM pain, and the activation of BKR induces endothelin-1 in endometriosis lesions, and that can cause pain [20].

In our study, the presence of adenomyosis is another independent risk factor for the recurrence of endometrioma. Sun Man et al. [21] found that extrinsic adenomyosis was significantly correlated with the early recurrence of endometrioma. Libo Zhu et al. found that the recurrence rate of AM combined with EM at 6 years after operation was higher than that of AM alone group [22], indicating that EM and AM influenced each other. However, some studies found that the effect of AM on the recurrence of endometrioma was not statistically significant [15].

Older age at surgery is an independent protective factor for endometrioma recurrence after surgery in our study. Several studies came to the same conclusion: younger age at surgery was a risk factor for recurrence [16, 23, 24]. Meta-analysis showed that younger age was a high risk factor for recurrence of endometrioma after conservative surgery [15, 25]. Nozomi Ouchi et al. analyzed patients with untreated endometrioma after surgery and found that age < 32 years old is a risk factor for postoperative endometrioma recurrence [26]. Some studies have found that age < 40 is an independent prognostic factor for EM recurrence [27]. EM is a hormone-dependent disease, estrogen and progesterone resistance are key events that caused ectopic implantation of endometrial cells and reduced the apoptosis as well as increasing oxidative stress, inflammation and neuroangiogenesis [28]. It can be argued that the relatively high estrogen level in young EM patients increases the possibility of the recurrence of endometrioma.

It should be noted that univariate logistic regression analysis revealed that family history of endometriosis or adenomyosis may be another risk factor for recurrence of endometrioma. The incidence of EM has a familial tendency [29]. Sisters with EM have an increased familial risk of IRR 2.75 (95%CI 2.25–3.36) compared with sisters without EM, with twins having the highest risk [30]. Sebastiano Campo etc. [31] found that family history of endometriosis is the only independent risk factor of postoperative endometrioma recurrence. However, we did not find the difference in the multivariate analysis in our study.

In addition, in our study, we could not find any significant differences when comparing patients with or without recurrence in terms of BMI, educational background, age of menarche, gravida, parity, leiomyoma, or postoperative use of GnRH-a. But some studies have come to different conclusions. It is reported that postoperative drug therapy could reduce postoperative recurrence of ovarian endometriotic cyst [32, 33]. Meta-analysis also showed that, after an average follow-up of 29 months, the recurrence rate of patients with endometrioma treated with dienogest was only 2%, while after an average follow-up of 36 months, the recurrence rate was 29% [34]. A retrospective single-center study involving 408 cases of endometrioma aged 40–49 years found that postoperative medical treatment could not reduce the recurrence rate [13].

Some studies found other possible risk factors that influencing the recurrence of endometrioma. Ovarian preservation was an independent risk factor for postoperative recurrence of endometrioma over 45 years old [35]. The depth of endometrial tissue infiltration into the cyst wall was a risk factor for endometrioma recurrence, and the optimal cut-off value was 1.2 mm, at which time the sensitivity was 62.9% and the specificity was 75% [36]. Large cyst size was another risk factor for recurrence in EM patients aged 40–49 years [13]. Postoperative pregnancy was another protective factor to reduce postoperative recurrence of ovarian endometriotic cyst [32]. The study result of Moini et al. showed that high rASRM score and large cyst are important factors for the recurrence of endometriotic cyst [24]. All in all, the recurrence of endometrioma is influenced by many factors and the mechanism of this is complicated. Hormonal, neurological and immune factors are involved in the mechanism of promoting the development of the disease [8]. Some studies have also found that the mechanism of endometriosis recurrence may be related to the overexpression of LncRNA H19 [27].

Our research has the advantage of a wide range of questionnaire survey, which requires experienced gynecologists and a large number of samples However, there are still some limitations. This is a cross-sectional study, which may contain bias in this study on patients with regional characteristic. The sample size of this study was not large enough, and the determination of recurrence depended on the skill of the sonographers and the experience of the gynecologists. A large, high-quality, long-term follow-up study is needed in the future.

In conclusion, we conducted a cross-sectional study of 874 patients from 27 hospitals in China. Our study showed that the presence of dysmenorrhea and adenomyosis were independent risk factors for postoperative recurrence of ovarian endometriotic cysts, and age at surgery was a protective factor. Therefore, for young patients with dysmenorrhea and adenomyosis, they should be alert to the recurrence of ovarian endometriotic cyst after surgery. Long-term chronic disease management should be carried out to delay the recurrence. For young patients with no recent fertility requirements and no serious symptoms, surgery should be delayed as far as possible.

## Data Availability

The data used in this study will be available upon reasonable request from the senior author, Ruihua Zhao.
